# Editorial: Social and Psychological Determinants of Value Co-creation in the Digital Era

**DOI:** 10.3389/fpsyg.2021.683829

**Published:** 2021-05-28

**Authors:** Ricardo Martínez-Cañas, Maria Angeles García-Haro, Pablo Ruíz-Palomino, Louise Kelly

**Affiliations:** ^1^Business Management Department, University of Castilla-La Mancha, Cuenca, Spain; ^2^Economic and Business Studies, Open University of Catalunya, Barcelona, Spain; ^3^Business and Public Management, University of La Verne, La Verne, CA, United States

**Keywords:** value co-creation, organizational outcomes, organizational contingencies, social determinants, psychological determinants

Organizations in the twenty-first century continuously adapt their mindsets and look for new ways to reawaken employees' talent toward innovation and competitiveness. In this regard, they fulfill new environment requirements together with more effective and interactive competitive strategies such as: fostering cooperation, promoting relationships of resource/competencies exchange, and developing networks through communication platforms and virtualization. There is a constant transformation of administrative processes from isolated into interactive processes. Consequently, a more psychological and social approach is needed to understand the real value and combined outcomes experienced through value co-creation activities. For a better understanding, organizational studies should consider a holistic view of stakeholders and their context as co-creators of the key-value needed to excel and be competitive.

This Research Topic for Frontiers in Psychology brings together a set of contributions related to social and psychological determinants of value co-creation for organizations and individuals.

In a brief, value co-creation reflects the value created and experienced through collaboration between multiple stakeholders (Prahalad and Ramaswamy, 2000; Saarijärvi et al., 2013). This concept has become a paradigm shift highlighting joint outcomes of a service organization's interaction with stakeholders (Grönroos, 2017). Also, value co-creation is a mechanism of the business model that creates a unique strategic relationship (de Oliveira and Cortimiglia, 2017). Moreover, it can become the central focus of organizations wanting to create superior shared value (Merz et al., 2018). However, the outcomes are affected by diverse internal and external determinants such as, individual/group/organizational characteristics, expectations, degree of participation, and environment (Jaakkola et al., 2015; Hsieh and Chang, 2016). Understanding the influence of these factors and contingent situations allows companies to strategically leverage their interactions with key stakeholders.

Recent, systematic review of the topic identified three broad research perspectives: psychological elements of value co-creation, content and processes, and innovation results (Fan and Luo, 2020). The present Special Issue brings together 12 studies by 53 authors who analyzed value co-creation and its relevance to addressing gaps of these research perspectives.

In the first, related to psychological elements of value co-creation, the research question is: why do customers participate in co-creation? Therefore, is value co-creation related to psychological factors such as motivation and personal characteristics? In this Research Topic:

Méndez-Aparicio et al. analyzed how customers' experience and satisfaction were related to value co-created with users of a private insurance corporation's web platform. Their empirical results showed that user's expectations were only relevant before web consumption.

Liu et al. explored the relationship between users' psychological contracts and their knowledge contribution in online health communities. Their results showed that users' transactional psychological contracts harmed their knowledge contribution directly and indirectly by weakening their community identification.

Carranza et al. studied how e-banking adoption could be an opportunity for customer value co-creation. Their results suggested that when e-banking customers had a positive attitude toward using e-banking they also had a greater intention to co-create and collaborate.

Hasan Al-kumaim et al. studied the role of sustaining continuous engagement through online platforms in value co-creation among individuals in universities. Their results proved that personal factors and perceived usefulness are motivational factors, however, the significance of these findings was contingent on individuals.

The second stream related to consumer processes is related to the question: how to carry out value co-creation?. The response includes activities on how to manage problems and elements in collaboration. In this Research Topic:

Siddique et al. theoretically studied customer engagement valence in a virtual service network. Their research analyzes how customer engagement's valence depends on the cognitive interpretation of positive signals (co-creating) and negative signals (co-destructing) prompted by multiple actors on a web store service network.

González-Santa Cruz et al. studied the internal marketing dimensions in cooperatives and social economy organizations. Their research about Ecuador's cooperative phenomenon highlighted the importance of providing quality service content to the external customer's characteristics and needs.

Garro-Abarca et al. analyzed virtual teams' role in times of pandemic, exploring factors that influence performance. Their paper groups the main elements into different models proposed by the literature and analyzes a study conducted amid the Covid-19 crisis on 317 software development teams. They found that communication processes are crucial to working in virtual teams, and also trust management is essential for leadership, empowerment, and cohesion.

Finally, in the research stream related to the impact results of value co-creation, variables related to innovation, engagement, loyalty, among other aspects, will have positive outcomes. In the Research Topic:

González-Torres et al. used a service-dominant logic perspective and a bibliometric analysis for analyzing the value co-creation effect of industrial clusters and global value chains. Their research remarked the critical change of these systems from the traditional isolated configuration to a more co-produced value chain structure with specialized interactive services.

Nájera-Sánchez et al. mapped value co-creation literature in the technology and innovation management field. They found eleven thematic groups and obtained significant research streams such as open innovation, consumer-centric analysis, service ecosystem, and service innovation. Also, they identified two new trends in literature: servitization and the sharing economy phenomenon.

Arroyave et al. analyzed the importance of universities' cooperation activities with different agents for building value co-creation systems for obtaining eco-innovations and improving firm performance. Using a sample of 250 companies in Spain, they found that value co-creation eased operational flexibility and generated environmental innovations and therefore increased the firm's sales and benefits.

Ortiz et al. studied the mediating role of realized absorptive capacity in the relationship between intra-organizational social capital and product innovation. Their results suggested that strong and tightly knit links based on a shared understanding and trust among company members lead the firm to develop dynamic capabilities.

Jiménez-Zarco et al. examined female micro-entrepreneurs and social networks for diagnostic analysis of social media marketing strategies' influence on brand financial performance. Using an online survey, they found that using social media tools in marketing actions significantly affects economic performance.

Given this selection of papers, we encourage researchers to draw their conclusions about value co-creation's complex phenomena. Our hope as editors of this Special Issue is that these different perspectives will encourage a prompt discussion among Journal Frontiers in Psychology readers.

## Author Contributions

All authors listed have made a substantial, direct and intellectual contribution to the work, and approved it for publication.

## Conflict of Interest

The authors declare that the research was conducted in the absence of any commercial or financial relationships that could be construed as a potential conflict of interest.
